# Solutions to improve the latent tuberculosis Cascade of Care in Ghana: a longitudinal impact assessment

**DOI:** 10.1186/s12879-020-05060-0

**Published:** 2020-05-18

**Authors:** Leila Barss, Joseph Obeng, Federica Fregonese, Olivia Oxlade, Benjamin Adomako, Anthony Opoku Afriyie, Erica Dapaah Frimpong, Nicholas Winters, Chantal Valiquette, Dick Menzies

**Affiliations:** 1 McGill International TB Centre, Montreal, QC Canada; 2 Komfo Anoyke Teaching Hospital, Kumasi, Ghana; 3 Respiratory Epidemiology and Clinical Research Unit, 5252 Boul. de Maisonneuve Ouest, Office 3.58, Montreal, QC H4A 3S5 Canada

**Keywords:** LTBI, Improvement, End TB strategy, Cascade of care

## Abstract

**Background:**

Loss of patients in the latent tuberculosis infection (LTBI) cascade of care is a major barrier to LTBI management. We evaluated the impact and acceptability of local solutions implemented to strengthen LTBI management of household contacts (HHCs) at an outpatient clinic in Ghana.

**Methods:**

Local solutions to improve LTBI management were informed by a baseline evaluation of the LTBI cascade and questionnaires administered to index patients, HHCs, and health care workers at the study site in Offinso, Ghana. Solutions aimed to reduce patient costs and improve knowledge. We evaluated the impact and acceptability of the solutions. Specific objectives were to: 1) Compare the proportion of eligible HHCs completing each step in the LTBI cascade of care before and after solution implementation; 2) Compare knowledge, attitude, and practices (KAP) before and after solution implementation, based on responses of patients and health care workers (HCW) to structured questionnaires; 3) Evaluate patient and HCW acceptability of solutions using information obtained from these questionnaires.

**Results:**

Pre and Post-Solution LTBI Cascades included 58 and 125 HHCs, respectively. Before implementation, 39% of expected < 5-year-old HHCs and 66% of ≥5-year-old HHCs were identified. None completed any further cascade steps. Post implementation, the proportion of eligible HHCs who completed identification, assessment, evaluation, and treatment initiation increased for HHCs < 5 to 94, 100, 82, 100%, respectively, and for HHCs ≥5 to 96, 69, 67, 100%, respectively. Pre and Post-Solutions questionnaires were completed by 80 and 95 respondents, respectively. Study participants most frequently mentioned financial support and education as the solutions that supported LTBI management.

**Conclusion:**

Implementation of locally selected solutions was associated with an increase in the proportion of HHCs completing all steps in the LTBI cascade. Tuberculosis programs should consider prioritizing financial support, such as payment for chest x-rays, to support LTBI cascade completion.

## Background

Latent tuberculosis infection (LTBI) management is a key component in the World Health Organization (WHO) End TB Strategy [1]. Household contacts (HHCs) of individuals with active pulmonary tuberculosis (TB) have a high risk of developing active TB [2]. Recent WHO guidelines recommended LTBI treatment for all HHCs diagnosed with LTBI in both low and high incidence TB countries [2].

**Fig. 1:**
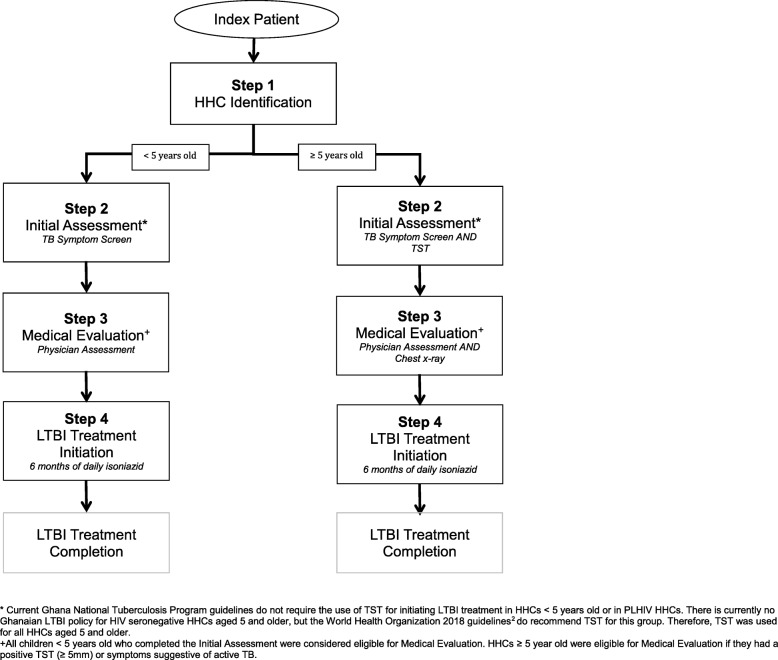
Latent Tuberculosis Cascade of Care at the study site in Ghana

Management of HHCs is a multistep process that begins with identification and concludes with treatment completion. This process has been referred to as the LTBI Cascade of care (Fig. 1). Recent systematic reviews have identified substantial patient losses at steps throughout the LTBI Cascade [3, 4]. The largest proportion of losses occurs prior to LTBI treatment initiation, but few studies have examined reasons, or evaluated solutions for these losses [3].

Ghana is a high incidence TB country, with an estimated incidence rate of 152 per 100,000 population in 2017 [5]. TB care in Ghana is provided through the Ghana National Tuberculosis Program (NTP). The 2015–2020 NTP strategic plan identified provision of isoniazid preventative treatment to high risk HHCs as a key activity [6]. There is no published evaluation of the implementation of these targets in practice in Ghana [5].

A multi-centre cluster randomized trial of a programmatic public health intervention to improve LTBI treatment initiation in HHCs was completed in 2019 (Enhancing the Public Health Impact of LTBI Diagnosis and Treatment (ACT4) [7]). As part of this study local stakeholders selected and implemented solutions to improve LTBI management based on local LTBI cascade and questionnaire data.

We have evaluated the impact and acceptability of the locally implemented solutions to strengthen LTBI management at the study site in Ghana. Specific objectives were to: 1) Compare the proportion of eligible HHCs completing each step in the LTBI cascade of care before and after implementation of the local solutions; 2) Compare knowledge, attitude, and practices (KAP) related to LTBI management before and after implementation of the solutions, based on responses of patients and health care workers (HCW) to questionnaires; 3) Evaluate patient and HCW acceptability of solutions using information obtained from these questionnaires.

## Methods

### Study design

#### Overview of ACT4 trial

ACT4 was a pragmatic cluster randomized trial at 24 sites in 5 countries. The full protocol has been published elsewhere [7]. Intervention sites received a two-phase intervention. In Phase 1, sites performed a standardized evaluation to identify and understand local gaps and barriers to LTBI care for HHCs of pulmonary TB patients. This included an evaluation of the LTBI cascade of care at each site, and questionnaires that were administered to Patients (index patients, adult HHCs, parents of child HHCs) and HCW. Site-specific solutions were then developed in consultation with local stakeholders. The solutions at the Ghana study site, summarized in Table 1, targeted barriers related to knowledge, stigma, and patient cost. In Phase 2, sites implemented their solutions with financial support provided by the research trial.

**Table 1:** Description of the Solutions Implemented at the Ghana Study Site

	Description	Cascade Step affected^a^
Program Strengthening^b^		
Initial and in-service health care worker training	• Initial training-LTBI management training sessions for HCW (cascade of care steps, TST administration and reading, INH administration). These sessions also included a review of data entry/management using the LTBI contact registry. Two full days sessions were held. Approximately 20 people present.	All steps
	• Initial training-Implementation of local solutions. One full day session. ~ 40 people present.	
	• In-service training (full day)- Weekly for the first 2 months; bi-weekly × 2 sessions; then monthly for remainder of Phase 2. These sessions included a review of data collection and entry into the registry, LTBI management, and an assessment of how the solutions were functioning.	
Solutions		
Educational materials	• Information posters about LTBI diagnosis and treatment in HHCs were created.	All steps
	• Posters were put up through the clinic waiting room and doctor’s offices.	
Phone reminders	• HCW were provided with phone vouchers to cover the cost of calling patients for visit reminders and follow-ups. A call was made to every contact before their visit and a follow-up call was made to all HHCs after they initiated treatment.	Step 1
		Step 2
		Step 3
Community Education	• Series of group education sessions conducted by the community health team from the Offinso clinic (2 members/session) at local schools, churches, and mosques.	Step 1
	• Sessions focused on LTBI, contact investigation, and stigma reduction.	Step 2
	• A total of six sessions were conducted.	
Community leader education/de-stigmatization *(Durbar)*	• A large meeting with local chiefs and sub-chiefs, as well as community opinion leaders was held.	Step 1
	• TB and LTBI education was provided. The aim was to gain the support and trust from the attendees so that they would encourage local people to participate in LTBI screening, diagnosis, and treatment.	Step 2
Home visits	• Routine home visits to all newly diagnosed index patients were implemented. Two HCW would visit the index patient’s home within the first 2 weeks of diagnosis.	Step 1
	• At the visit, HHCs were identified and a symptom screen and TST (for those eligible) was performed.	Step 2
	• A home visit was also performed for all HHCs started on LTBI treatment (HCW would drop off LTBI medications and perform a monitoring visit).	
Patient transport reimbursement	• Patients were reimbursed for their transportation costs to the clinic. All types of visits were covered (initial assessment, treatment follow up, etc.)	Step 2
	• Patients were also given a per diem cost to cover the cost of lunch on the day of their medical evaluation clinic visit.	Step 3
Chest x-ray (CXR) reimbursement	• The cost of obtaining a CXR was covered for all contacts over 5 years old who had a positive TST. If a contact had medical insurance, the remaining cost not covered by insurance was covered (a minority of patients had insurance coverage). Insurance would cover 25 GHC (total cost of CXR is 40 GHC).	Step 3
WhatsApp group for physicians	• After the implementation of digital CXR in the region, a WhatsApp group for doctors was initiated to enable faster interpretation and feedback.	Step 4
	• Call vouchers were provided to doctors taking care of HHCs during the study to allow them to pay for data for this service.	

^a^Step 1-Identification of contacts; Step 2-Initial assessment; Step 3-Medical Evaluation; Step 4-Treatment Initiation. See Fig. 1 for detailed description of cascade steps for those < 5 years of age and ≥ 5 years of age

^b^Program strengthening activities were done in all study sites. Each site determined the LTBI educational content that was included in their healthcare worker training sessions. At the study site in Ghana, healthcare workers were educated about LTBI management for household contacts. Explicit training for how they should educate patients was not provided

#### Solutions impact study (see also Fig. 2: schematic of study design)

For this study we used a quasi-experimental design to compare the LTBI cascade of care before (April–June 2017; “Pre-solutions cascade”) and after solution implementation (February to April 2018;“Post-solutions cascade”). The Cascades included all HHCs of index patients diagnosed with microbiologically confirmed pulmonary TB. In an earlier study conducted in Ghana, no HHC completed any LTBI cascade steps past “Identification”, meaning that no HHCs were investigated, nor treated. Based on this, we calculated that the expected number of HHC identified for 6 index patients (*n* = 56 HHCs) Post Solutions would provide > 80% power to detect a 30% increase in Identification. Due to variability in the number of index patients identified per month, a three-month period was selected for the Pre and Post cascades to ensure an adequate sample size. Cascade data was collected for HHCs from Step 1, Identification, to Step 4, Treatment Initiation.

**Fig. 2:**
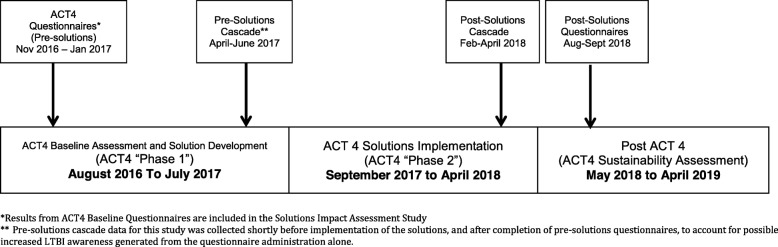
Timeline of ACT4 Study and Solutions Impact Assessment Study Data Collection

To assess solution acceptability, we used the results from the questionnaires administered between November 2016 to January 2017 to Patients and HCW as part of the ACT4 trial (“pre-solutions questionnaires”). In August and September 2018, we administered a second set of questionnaires to Patients and HCW (“post-solutions questionnaires”).

### Study setting

This study was conducted at St. Patrick’s Hospital Outpatient TB clinic in Offinso, Ghana between November 2016 and September 2018. St. Patrick’s hospital is part of the National Catholic Health Services system in Ghana. The hospital has a catchment area of approximately 90,000 people. In 2016 and 2017 there were approximately 58 patients treated for active TB each year. None of their HHCs were started on LTBI treatment during this time. At a national level, the Ghanaian NTP coordinates TB care. There are ten administrative regions in the country with Regional NTP Coordinators responsible for TB management in each region. TB care and services are provided free [6].

### Study participants

Index patients with newly diagnosed microbiologically confirmed pulmonary TB were eligible if they had at least one identified HHC. Adult HHCs and parents of child HHCs < 5 years of age were eligible to complete questionnaires if they (or their child) had been identified as a HHC of a patient with newly diagnosed microbiologically confirmed pulmonary TB. Index TB patients and HHC could complete only one questionnaire. Local HCW involved in routine patient care identified potentially eligible Patients. The TB clinic manager identified potentially eligible HCWs.

### Data collection

#### Cascade

The Index TB Patient register was used to identify all patients diagnosed with active pulmonary TB during all data gathering periods. Prior to solution implementation, the study site used a “Register of TB Contact Investigations” to list identified HHCs and any contact investigation activities. This registry was used to collect information for the pre-solutions cascade. A more detailed contact registry containing information about identification, symptom screen, TST, chest x-ray, and treatment initiation/completion was implemented in the study, and used to collect data for the post-solution cascade.

#### Questionnaires

Questionnaires assessed KAP related barriers to LTBI care and were based on those used in a previous study (available on request from authors) [8]. The specific questions within each questionnaire differed according to the group surveyed (index patients versus HHCs versus HCW). Responses to open ended questions were coded into common themes. All questionnaires were pre-tested for clarity. For the post-solutions questionnaires, shortened versions of the pre-solutions questionnaires addressing KAP were used. Open-ended questions evaluating the respondents’ perspectives on the solutions were added (available on request from authors). The pre-solutions questionnaires were shortened to remove redundant and non-informative questions based on structured content analysis and feedback from study sites. A trained research assistant administered all questionnaires with the exception of the post-solutions questionnaire for HCW, which was self-administered. Questionnaires were administered in the participants’ primary language (Twi). The self-administered HCW questionnaire was in English. We aimed to interview 20 persons from each of the participant groups in the pre and post-solutions questionnaires.

### Analysis

The proportion of HHCs who successfully completed each cascade step among those eligible in the pre-solutions versus post-solutions assessment period was compared using Fisher’s exact test with *p* < 0.05 considered significant. We could not find any published data from Ghana estimating the number of expected HHCs per index patient. Therefore, we used data on the average number of HHCs identified per index TB patient in the last 3 months of the study, when the contact investigation procedures were well established. This number was then used to calculate the expected number of HHCs (i.e. those eligible to enter Step 1), for all study periods.

Questionnaire KAP responses on pre versus post-solutions questionnaire were compared using Fisher’s exact test with *p* < 0.05 considered significant.

Acceptability of solutions was based on post-solution questionnaires responses. Questions were open ended and respondents were not prompted regarding potential solutions, so they could mention literally any possible item. Therefore, if at least 30% of patient respondents listed the same solution as helpful, this solution was considered to have “good” acceptability. Mention of any item by at least one respondent was considered to represent “moderate” acceptability. The same criteria were used for HCW, with the exception that if a solution was reported as “most helpful” it was judged to have “good” acceptability. Data analysis was performed using statistical package for social science (SPSS) version 25.

### Ethics

This study was approved by McGill University Health Centre Research Institute ethical review board (ERB) (15–291-MUHC) and the Committee on Human Research, Publication and Ethics of the School of Medical Sciences/Komfo Anokye Teaching Hospital.

## Results

### LTBI Cascade completion

**Fig. 3:**
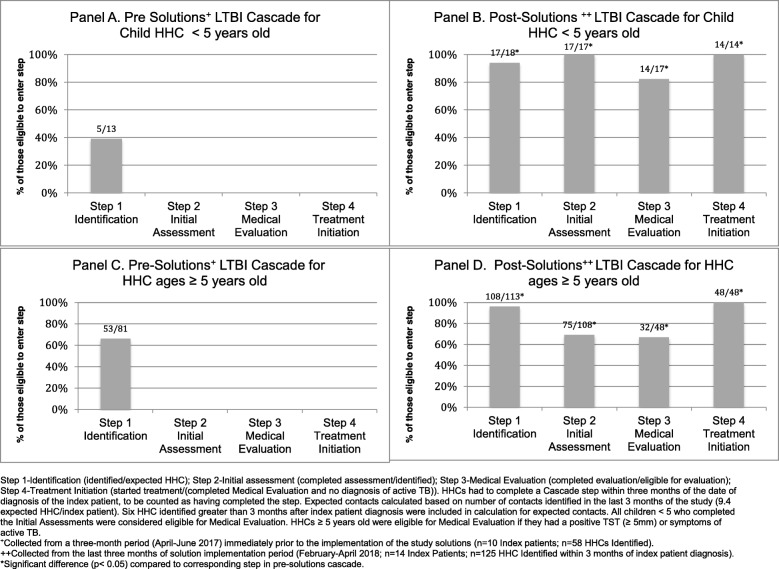
Pre and Post Solutions LTBI Cascade of Care for HHC: Children < 5 and all others ≥5 years old

As shown in Fig. 3, in the pre-solutions cascade, 39% of expected HHCs < 5 years (*n* = 5), and 66% of expected HHCs ≥5 years (*n* = 53), were identified. Medical assessment and LTBI treatment were not provided to any eligible HHCs. In the post-solutions cascade, the proportion of adult and child HHCs completing each step in the LTBI Cascade increased by at least 30%, compared to the pre-solutions cascade (*p* < 0.05 for all steps).

### Questionnaires: to assess the acceptability of the solutions

i. Characteristics of respondents

The characteristics of the 80 respondents who completed questionnaires before, and the 95 respondents who completed questionnaires after solution implementation were similar (Table 2).

ii. Patient and HCW Solution Acceptability

**Table 2:** Demographic Characteristics of Questionnaire Respondents

	Pre-solutions		Post-Solutions	
	n	%	n	%
**Adult Contacts**				
Number	20		30	
Age in yrs., median (range)	46.5 (19; 74)		35 (18; 85)	
Female Gender	12	60%	21	70%
Phase of Cascade				
Identified (no testing done)	20	100%	3	10%
Completed symptom screen +/− TST	–	–	12	40%
Medical investigations in-progress/completed	–	–	0	0
Recommended LTBI treatment	–	–	15	50%
**Parents of child contacts**				
Number	20		19	
Age of parent in yrs., median (range)	37.5 (23; 71)		32 (18; 44)	
Age of youngest child in yrs., median (range)	2 (1; 5)		3 (1; 5)	
Phase of Cascade				
Identified (no testing done)	20	100%	1	5%
Completed symptom screen +/− TST	–	–	6	32%
Medical investigations in- progress/completed	–	–	0	0
Recommended LTBI treatment	–	–	12	63%
**Index Patients (with active TB)**				
Number	20		16	
Age in yrs., median (range)	38.5 (23; 78)		42.5 (21; 80)	
Female gender	5	25%	4	25%
**Health care workers**				
Number	20		25*	
Job Title				
Doctor	0	0%	1	4%
Nurse	17	85%	18	72%
Other	3	15%	6	24%

*2 health care workers completed both a pre and post solutions questionnaire

Home visits (9/12(75%)) and education (5/12(42%)) were the activities that HCWs most frequently reported as helpful for Identification of HHCs.

**Fig. 4:**
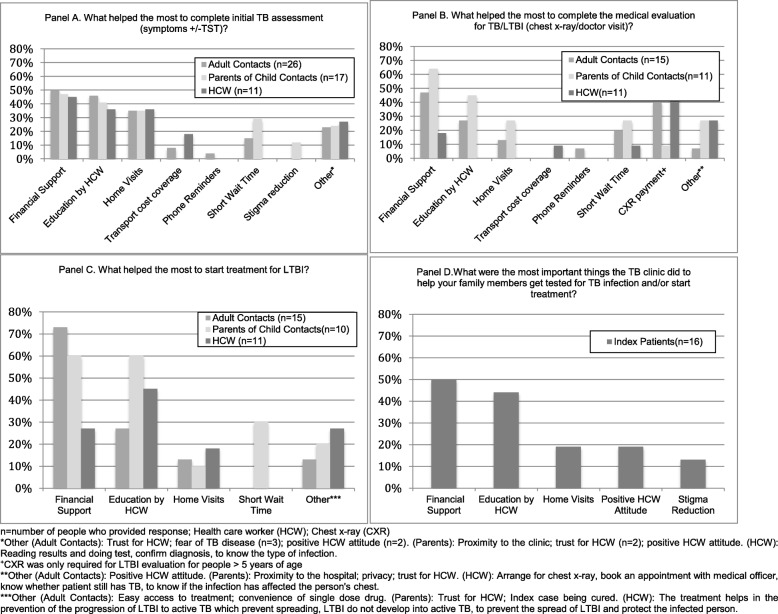
Acceptability and benefits of Solutions implemented during study - Patient and Health Care Worker Assessments from the Post-Solutions Questionnaires

As shown in Fig. 4, financial support, education provided by HCW to HHCs, and home visits were the most frequently reported determinants that supported completion of the Initial Assessment by Patients and HCWs.

In the post-solutions questionnaires, adult HHCs (7/15(47%)), parents of child HHCs (7/11(64%)), and HCW (2/11(18%)) all reported that financial coverage was an important determinant in completing the Medical Evaluation (Fig. 3). Both adult HHC (6/15(40%)) and HCW (4/11(40%)) specifically mentioned financial coverage of the cost for chest x-ray was important. Education provided by HCW was also mentioned as an important determinant by adult HHC (3/11(27%)) and parents of child HHC (5/11(45%)).

Financial support and education provided by HCW were the most frequently reported determinants by HHCs and HCW that supported LTBI treatment initiation.

A summary of the patient and HCW solution assessments, along with a description of solutions adoption at the study conclusion, is shown in Table 3. HCW training, educational materials, home visits, and chest x-ray funding were all reported to have good Patient (index patient, HHC, and parent of child HHC) and provider (HCW) acceptability (Fig. 4). The main determinant of successful adoption of a solution was the availability of funding for the solution (e.g. reimbursement for the cost of a chest x-ray had good patient and provider acceptability but was not adopted because funding could not be obtained).

iii. Knowledge, attitude, and practices

**Table 3:** Patient and Provider judged Acceptability of Solutions (based on Post-Solutions questionnaire) and post study adoption

Solutions	Acceptability^a^		Post study adoption
	Patient	Provider	
Program Strengthening			
Initial and in-service HCW training^b^	Good	Good	Yes
			*Program Funded*
Local Solutions			
Educational materials	Good	Good	Yes
			*Program Funded*
Phone reminders	Moderate	Moderate	Yes
			*Self funded (HCW)*
			HCW have continued to make reminder and follow-up phone calls but they are paying for the cost out of their own pockets.
Community Education	Moderate	Not reported	Yes
			*Program Funded*
			The LTBI clinic health care workers have been able to join with other public health activities funded by the hospital to provide LTBI education at these sessions.
Community leader education/stigma reduction *(Durbar)*	N/A	N/A	No
Home visits	Good	Good	Yes
			*Program Funded*
			The hospital provided funding for a vehicle for transport to allow HCW to continue home visits.
Patient transport reimbursement	Moderate	Good	No
CXR reimbursement	Good	Good	No
			Funding for CXR reimbursement from the hospital could not be obtained. The clinic is currently recommending patients travel to another hospital where they can obtain CXR for free (patients pay for cost of travel).
WhatsApp for physicians	N/A	Not reported	Yes
			*Self funded (Physicians)*
			Physicians have continued to use this program but are paying for the data costs on their own.

Health care workers (HCW); Chest x-ray (CXR)

^a^Solutions were judged to be acceptable based on the responses in the post-solutions questionnaires. If ≥30% of Patient respondents (either adult HHCs, parents of child HHCs, index patients) listed a solution as helpful then the solution was considered to have “good” patient acceptability (Fig. 3). If between 3 and 30% of Patient respondents listed a solution as helpful, the solution was considered to have “moderate” acceptability. If solution was not directly assessed in the questionnaires, then “not applicable (N/A)” was reported. For HCWs, the same criteria were used, with the exception that if HCWs selected a solution as one of the “most helpful” solutions it was judged to have “good” acceptability (Fig. 4; Appendix 1-Table 3)

^b^No explicit training was provided to HCW regarding patient education, however, the initial and in-service training provided HCW with knowledge to educate patients, and therefore, for the analysis the response “HCW education to patients” was attributed to this solution

Detailed responses to each section of the questionnaires are presented in Supplemental Tables 1A-1E. The proportion of patients who reported they completed the Initial LTBI Assessment because of HCW advice in the post-solutions questionnaire significantly increased (5% versus 33% for pre versus post-solutions questionnaires respectively, *p* < 0.05) (Appendix 1-Supplemental Table 1A).

Pre-solutions, all parents reported their child did not complete a medical assessment for TB/LTBI because they lacked adequate knowledge (Appendix 1-Supplemental Table 1B). Post-solution, this was not reported as a barrier by any parents.

Overall, “affordability/low cost” was the most frequently reported reason for clinic satisfaction by parents of child HHCs and the third most frequently reported reason for clinic satisfaction by adult HHCs and index patients (Appendix 1-Supplemental Table 2).

## Discussion

In this assessment of strategies to improve LTBI management in Ghana, the implementation of locally selected solutions was associated with large improvements in the LTBI cascade. Financial support and patient education were the most frequently reported factors that supported LTBI cascade completion.

In low and middle-income countries, the cost of chest x-ray alone can be a major financial burden on patients with TB and their families [8, 9]. In this study, payment for chest x-rays was implemented as a solution to financially support HHCs. The average monthly salary prior to TB diagnosis among patients with active TB in Ghana has been estimated at approximately 300 GHC ($62 USD) [9]. At the time of the study, the local cost for one chest x-ray was 40 GHC ($10 USD). If two or three HHCs from one family require a chest x-ray for LTBI medical assessment, as is often the case, it is clear that the cost for this step alone could be a major barrier.

Several studies of HHCs have reported significant knowledge gaps surrounding TB transmission, screening measures, and infection status [10–13]. The solutions at the Ghana site included education for HCW and patients. Patient education was consistently reported by HCW, and by the HHC themselves, as contributing to LTBI cascade completion.

Our study has several limitations. The lack of an external control group for comparison limits our ability to attribute the improvements in the cascade to the study solutions. However, no other changes were made to the LTBI management program during this time so the impact of other or external factors should have been minimal. Secondly, our data is limited to a single site. Sample size calculation was based only on Step 1- Identification; however, given the large improvements at all steps this likely has minimal impact on interpretation of our results. Post-solutions questionnaires were administered after some solutions had been discontinued. Therefore, some respondents may not have been exposed to all solutions, thereby limiting their assessment. Post-solutions HCW questionnaire was self-administered. This likely reduced response bias, however, some respondents misinterpreted some questions, which limited the number of informative responses. Finally, we were not able to assess possible interaction effects of different program strengthening activities and solutions on each other.

Based on our results, reducing the financial burden for HHC should be a key priority for the Ghanaian NTP. Education of the index TB patients and their HHCs should also be a priority. Finally, despite improvements in LTBI cascade completion, some gaps persisted at the study site. In order to continuously improve TB programs, repeated assessments of the LTBI cascade are needed.

## Conclusion

Following the implementation of multiple solutions to strengthen LTBI management at a TB clinic in Ghana, there was a large increase in the proportion of HHC started on LTBI treatment. Although inferences are limited from a single site study, our conclusion is that reducing financial barriers and providing education to index patients and HHCs were the most important solutions.

## Supplementary information

**Additional file 1 Appendix 1.** Supplemental Tables. Contains questionnaire results not included within main manuscript.

## Data Availability

All relevant data generated and analysed during the current study are included in this published article (and it supplementary information files). Further data that was considered non-relevant (limited questionnaire data) are available from the corresponding author on reasonable request.

## Abbreviations

ACT4: Enhancing the Public Health Impact of LTBI Diagnosis and Treatment
GHC: Ghanian Cedis
HCW: Health Care Worker
HHC: Household Contact
LMIC: Low and Middle Income Countries
LTBI: Latent Tuberculosis Infection
TST: Tuberculin Skin Test
TB: Tuberculosis
USD: United States Dollar
WHO: World Health Organization
