# Supplementary material for: Solutions to improve the latent tuberculosis Cascade of Care in Ghana: a longitudinal impact assessment
Source: BMC Infect Dis. 2020 May 18;20:352. doi: 10.1186/s12879-020-05060-0 (PMC7236456; doi:10.1186/s12879-020-05060-0)
Supplement: Supplementary file 1 — Additional file 1 Appendix 1. Supplemental Tables. Contains questionnaire results not included within main manuscript. [file 12879_2020_5060_MOESM1_ESM.docx]

Supplemental Table 1. Comparison of Pre-Solutions and Post-Solutions Responses:

Supplemental Table 1A. Related to Initial Assessment

|  | Pre-solutions | | Post-Solutions | | |
| --- | --- | --- | --- | --- | --- |
|  | n | % | n | % | |
| **Adult Contacts** | n=20 |  | n=30 |  | |
| **Did you go (or are you planning to go) to the clinic/hospital to be checked or tested for TB?** | | | | | |
| Yes | 16* | 80% | 19^A^ | 63% | |
| No | 4 | 20% | 11 | 37% | |
| **If "Yes", why?** | | | | | |
| Worried/ concerned about personal health/fear of TB^+^ | **14** | **70%** | **3** | **10%** | |
| For prevention/ health maintenance | 12 | 60% | 14 | 47% | |
| Advice of health care worker^+^ | **1** | **5%** | **10** | **33%** | |
| Felt sick, had symptoms, needed to be checked/ tested | 1 | 5% | 1 | 3% | |
| Advice of friends or family or co-workers (to go) | 0 | 0% | 1 | 3% | |
| **If "No", why?** | | | | | |
| Do not need, do not believe, not a priority/I am not sick/I have no symptoms of TB^+^ | **4** | **20%** | **0** | **0%** | |
| Cost - for travel, testing, physician visit, time off work | 0 | 0% | 3 | 10% | |
| Too busy, could not take time off, would take too much time | 0 | 0% | 2 | 7% | |
| Not convenient - location, time, hours of operation, long wait times | 0 | 0% | 2 | 7% | |
| Advice of family or friends or co-workers (to not go) | 0 | 0% | 1 | 3% | |
| Other** | 0 | 0% | 4 | 13% | |
| **Parents of Child Contacts** | n=20 |  | n=19 |  | |
| **Did you take your child (or are you planning to take your child) to the clinic/hospital to be checked or tested for TB?***** | | | | | |
| Yes | 16 | 80% | 17 | 89% | |
| No | 4 | 20% | 2 | 11% | |
| **If "Yes", why?** | | | | | |
| For prevention/ health maintenance | 14 | 70% | 12 | 63% | |
| Worried/ concerned about personal health/fear of TB | 9 | 45% | 4 | 21% | |
| Advice of health care worker^+^ | **5** | **25%** | **12** | **63%** | |
| **If "No", why?** | | | | | |
| Afraid of stigma or discrimination | 4 | 20% | 0 | 0% | |
| Do not need, do not believe, not a priority/ Child is not sick/Child has no symptoms of TB | 3 | 15% | 1 | 5% | |
| Cost - for travel, testing, physician visit, time off work | 0 | 0% | 1 | 5% | |
| **Index Patients** | n=20 |  | n=16 |  | |
| **Did someone at the clinic explain to you that your household members should be checked or tested for TB?** | | | | | |
| Yes^+^ | **13** | **65%** | **16** | **100%** | |
| No | 7 | 35% | 0 | 0% | |
| **Did you encourage *all* of the members of your household to be checked or tested for TB?** | | | | | |
| Yes | 17 | 85% | 16 | 100% | |
| No | 3 | 15% | 0 | 0% | |
| **If "Yes", why?** | | | | | |
| Protect others. Protect family / friends / co-workers. Prevent transmission | **13** | **65%** | **3** | | **19%** |
| Advised and believe doctor or nurse or other worker. Trust doctor/nurse. | 7 | 35% | 10 | | 63% |
| Fear, worried, concerned about TB, concerned about sickness | 5 | 25% | 9 | | 56% |
| Protect their health, stay healthy, prevent illness | 5 | 25% | 4 | | 25% |
| **If "No", why?** | | | | | |
| I did not know TB was contagious | 2 | 10% | 0 | | 0% |
| I did not know the clinic offered testing for household contacts | 1 | 5% | 0 | | 0% |
| **Health Care Workers** | **n=20** |  | **n=25** |  | |
| **Sometimes parents of young children do not bring their children to be screened/tested. Why do you think this happens?** | | | | | |
| Parents do not understand / have not been educated / denial / do not believe/ not aware/lack of knowledge^+^ | **13** | **65%** | **7** | | **28%** |
| Parents are afraid of stigma, discrimination for them/their child^+^ | **7** | **35%** | **0** | | **0%** |
| Parents does not believe they are sick / no symptoms of TB | 6 | 30% | 9 | | 36% |
| Cost for parents- for travel, testing, physician visit, time off work/school, parking | 3 | 15% | 9 | | 36% |
| Not convenient for parents/not accessible - location, time, hours of operation | 3 | 15% | 2 | | 8% |
| Fear of: treatment/side effects of treatment/harms of treatment/dislike antibiotics/pills/ blood tests/needle | 2 | 10% | 1 | | 4% |
| Parents are too busy, can't take time off, would take too much time/ would miss work or school for children, waiting time too long | 1 | 5% | 2 | | 8% |
| The HCW’s opinion is that "children don’t need to be screened anyway" | 1 | 5% | 1 | | 4% |
| The HCW’s opinion is that they DO bring their children (it’s not a problem) | 1 | 5% | 0 | | 0% |
| Other**** | 0 | 0% | 2 | | 8% |

^+^Result shown in bold indicate differences in responses between pre and post-solutions questionnaires, that are statistically significant with, p< 0.05.

*Pre-solutions question “Were you checked/tested for TB following the TB diagnosis of the index patient in your household?” (Yes, n=1) was combined with question “If no, will you go to be checked or tested soon?” (Yes, n=15).

^A^This question was used to refer to the Initial Assessment and the Medical Evaluation steps in the post-solutions questionnaires. 27/30 respondents had completed the Initial assessment (based on review of medical records), however, only 19/30 responded, “Yes” to this question.

**Pregnancy n=2; recently identified n=1; didn't meet staff at clinic n=1

*** Pre-solutions question “Was your child checked for TB using TST/IGRA following the TB diagnosis of the index case?” (n=1) was combined with question “If no, will you bring your child to be checked soon?” (n=15).

****Children are more prone to picking infections from touching and playing with items during visits to the hospital; bad attitude of the nurses.

Supplemental Table 1B: Related to Medical Evaluation for Parents of Child HHC*

|  | Pre-solutions | | Post-Solutions | | |
| --- | --- | --- | --- | --- | --- |
|  | n | % | n | % | |
| **Parents of Child Contacts** | **20** |  | **19** |  | |
|  | **Did your child have a medical evaluation at the clinic/hospital?** | | **Did you take your child (or are you planning to take your child) to the clinic/hospital to be checked or tested for TB/LTBI?**** | | |
| Yes^+^ | **0** | **0%** | **17** | | **89%** |
| No | 14*** | 70% | 2 | | 11% |
| **If "Yes", why?** | | | | | |
| For prevention/ health maintenance | - | - | 12 | | 71% |
| Advice of health care worker | - | - | 12 | | 71% |
| Worried/ concerned about personal health, fear of TB | - | - | 4 | | 24% |
| **If "No", why?** | | | | | |
| Was not told, not informed, did not understand, did not know where to go^+^ | **14** | **70%** | **0** | | **0%** |
| Do not need, do not believe, not a priority/Child was not sick/I child had no symptoms of TB | 3 | 15% | 1 | | 5% |
| Afraid of stigma or discrimination | 1 | 5% | 0 | | 0% |
| Cost - for travel, testing, physician visit, time off work | 0 | 0% | 1 | | 5% |

*Comparison of pre and post solution responses for Medical Evaluation for Adult HHC was not performed because only one adult HHC responded to the question in the pre-solutions questionnaire assessing Medical Evaluation

^+^Result shown in bold indicate differences in responses between pre and post-solutions questionnaires, that are statistically significant with, p< 0.05.

**In the post-solutions questionnaires this question was used to assess completion of the Initial assessment and Medical evaluation steps. Therefore, the responses to this question are also included in the main text Table 2 to allow for a comparison with the pre-solutions Initial assessment question responses.

***Only 14 Parents of child HHC responded to this question

Supplemental Table 1C. Related to Treatment Initiation*

|  | Pre-solutions | | Post-Solutions | |
| --- | --- | --- | --- | --- |
|  | n | % | n | % |
| **Adult Contacts** | **20** |  | **30** |  |
| **If the doctor/nurse advised you to take medication to *prevent* TB did you agree to take it (or, if they advise you to take medications to prevent TB, would you agree to take it)?** | | | | |
| Yes | ** |  | 28 | 93% |
| No | ** |  | 2 | 7% |
| **If "Yes", why?** |  |  |  |  |
| For prevention/ health maintenance | - |  | 20 | 67% |
| Worried/concerned about personal health, fear of TB | - |  | 10 | 33% |
| Protect myself, protect my own health, stay healthy, prevent illness | - |  | 8 | 27% |
| Protect others (family / friends /co-workers) from getting TB | - |  | 4 | 13% |
| Believe/trust doctor, nurse, or other health worker | - |  | 3 | 10% |
| **If "No", why?** |  |  |  |  |
| Cost - for travel, time off work | - |  | 1 | 3% |
| Fear of side effects of treatment, dislike antibiotics/pills, fear blood tests | - |  | 1 | 3% |
| **Parents of Child Contacts** | **20** |  | **19** |  |
| **If the doctor/nurse advised your child to take medication to *prevent* TB did you agree for your child to take it (or, if they advise your child to take medications to prevent TB, would you agree)?** | | | | |
| Yes | ** |  | 19 | 100% |
| No | ** |  | 0 | 0% |
| **If "Yes", why?** |  |  |  |  |
| For prevention/ health maintenance | - |  | 18 | 95% |
| Believe/trust doctor, nurse, or other health worker | - |  | 4 | 21% |
| Protect my child's health, make my child stay healthy, prevent illness | - |  | 4 | 21% |
| Worried/concerned about child's health, fear of TB | - |  | 2 | 11% |
| Protect others (family / friends /co-workers) from getting TB | - |  | 2 | 11% |
| **Index Patients** | **20** |  | **16** |  |
| **Did any of your household contacts start preventative treatment for TB?** | | | |  |
| Yes | - |  | 13 | 81% |
| No | - |  | 3 | 19% |
| **If "Yes", why?** |  |  |  |  |
| Advised and believe doctor/nurse/other worker | - |  | 9 | 56% |
| Protect themselves from TB, stay healthy, avoid getting TB/reduce the risk of TB | - |  | 6 | 38% |
| To protect others: prevent TB transmission | - |  | 6 | 38% |
| Fear, worried, concerned about TB, concerned about sickness | - |  | 3 | 19% |
| Treatment is effective and safe | - |  | 1 | 6% |
| **If "No", why?** |  |  |  |  |
| Cost - for travel, time off work | - |  | 3 | 19% |
| Cost - for testing, physician visit, medication | - |  | 1 | 6% |
| **Health Care Workers** | **20** |  | **25** |  |
| **If you were a household contact of a patient with active TB and you had a positive TST, would you take treatment for LTBI?** |  |  |  |  |
| Yes, I would definitely take treatment | 15 | 75% | 25 | 100% |
| Only if strongly advised | 4 | 20% | - | - |
| No | 1 | 5% | 0 | 0% |

*Comparison of pre and post solution responses for Treatment Initiation for Index Patients, Adult HHC, and Parent of Child HHCs was not performed because no respondents provided answers to these questions

^+^Result shown in bold indicate differences in responses between pre and post-solutions questionnaires, that are statistically significant with, p< 0.05.

Supplemental Table 1D:Related to General Knowledge

|  | Pre-solutions | | Post-Solutions | |
| --- | --- | --- | --- | --- |
|  | n | % | n | % |
| **Adult Contacts** | **20** |  | **30** |  |
| **How do you think TB is transmitted from one person to another?** | | | |  |
| Indirect contact - living in same house or in same room/eating meals together/ breathing the same air | 15 | 75% | 27 | 90% |
| Indirect contact – (through objects) – same dishes/cups/toothbrush | 5 | 25% | 9 | 30% |
| Direct contact with someone with TB disease | 2 | 10% | 5 | 17% |
| Not taking care of yourself: Becoming too tired/exhausted/poor diet | 0 | 0% | 1 | 3% |
| Does not know | 3 | 15% | 0 | 0% |
| Parents of Child Contacts | 20 |  | 19 |  |
| Indirect contact (through air) - living in same house or room / breathing the same air | 16 | 80% | 16 | 84% |
| Indirect contact –(through objects) - same dishes / cups / toothbrush | 2 | 10% | 7 | 37% |
| Direct contact with someone with TB disease | 6 | 30% | 6 | 32% |
| Unclean living environment | 0 | 0% | 2 | 11% |
| Sharing a razor | 1 | 5% | 0 | 0% |
| Index Patients | 20 |  | 16 |  |
| Indirect contact - living in same house or in same room/eating meals together/ breathing the same air | 19 | 95% | 16 | 100% |
| Indirect contact – (through objects) – same dishes/cups/toothbrush | 2 | 10% | 2 | 13% |
| Direct contact with someone with TB disease | 4 | 20% | 7 | 44% |
| Bad habits / unhealthy lifestyle / smoking / drinking alcohol / drugs | 1 | 5% | 0 | 0% |
| Unclean living environment | 1 | 5% | 0 | 0% |
| Health care workers | 20 |  | 25 |  |
| Indirect contact - living in same house or in same room/eating meals together/ breathing the same air | 20 | 100% | 24 | 96% |
| Indirect contact – (through objects) – same dishes/cups/toothbrush | 1 | 5% | 0 | 0% |
| Direct contact with someone with TB disease | 1 | 5% | 7 | 28% |
| Dust exposure | 0 | 0% | 3 | 13% |

^+^Result shown in bold indicate differences in responses between pre and post-solutions questionnaires, that are statistically significant with, p< 0.05.

Supplemental Table 1E: - Assessment of Financial Burden –Index tuberculosis patients

|  | Pre-solutions | | Post-Solutions | |
| --- | --- | --- | --- | --- |
|  | n=20 | % | n=16 | % |
| **Has having TB led to any financial problems or hardships in your family?** | | | | |
| Sold any personal belongings^+^ | **13** | **65%** | **1** | **6%** |
| Could not afford to buy food for themselves or family^+^ | **12** | **60%** | **2** | **13%** |
| Salary decreased/Using up savings/Took out a loan/ borrowed money (from bank or family or friends) ^+^ | **11** | **55%** | **0** | **0%** |
| Could not work as much/ prolonged time away from work/Lost your job/ worries about losing job | 6 | 30% | 9 | 56% |
| Could not pay rent, or had to change housing as could not afford current housing | 5 | 25% | 1 | 6% |
| Removed a child from school | 4 | 20% | 0 | 0% |
| Caused other major change in financial situation | 0 | 0% | 2 | 13% |
| No | 2 | 10% | 4 | 25% |

^+^Result shown in bold indicate differences in responses between pre and post-solutions questionnaires, that are statistically significant with, p< 0.05.

Supplemental Table 2. Satisfaction with Clinical Services – Patient responses from the Post-Solutions Questionnaire

|  | **Adult Contacts** | | **Parents of Child Contacts** | | **Index Patients** | |
| --- | --- | --- | --- | --- | --- | --- |
|  | N | % | n | % | n | % |
|  | 30 |  | 19 |  | 16 |  |
| **Can you mention anything that was good/worked well in this clinic?** | | | | | | |
| Trust staff | 22 | 73% | 8 | 42% | 13 | 81% |
| Good quality of care, well equipped | 16 | 53% | 8 | 42% | 10 | 63% |
| Free/ affordable/ low prices* | 11 | 37% | 12 | 63% | 10 | 63% |
| Convenience - location, time hours of operation, short wait time | 10 | 33% | 2 | 11% | 5 | 31% |
| It is a place I know (from visits in the past) | 2 | 7% | 0 | 0% | 1 | 6% |
| My family, friends, co-workers like this clinic | 1 | 3% | 0 | 0% | 1 | 6% |
| I have insurance which covers my care at this place | 0 | 0% | 1 | 5% | 0 | 0% |

“Free/ affordable/ low prices was indicated as a response, if the respondent mentioned any of these terms or mentioned a topic related to cost/finances.”

Supplemental Table 3. Assessment of Solutions –Health Care Workers responses from the Post-Solutions Questionnaire

| **From your perspective as a health care worker, what intervention was most helpful for you in your work taking care of patients with latent tuberculosis?**  **N=24 respondents** | | |
| --- | --- | --- |
|  | n | % |
| Education | 7 | 29% |
| Home Visits | 6 | 25% |
| Phone reminders | 4 | 17% |
| Free Cost | 3 | 13% |
| CXR Payment | 2 | 8% |
| Transport Payment | 1 | 4% |
| Other* | 13 | 54% |

*Other responses indicated that many HCW misinterpreted the question (Steps in the cascade were listed instead of solutions/strategies/interventions). Responses included: they are all very important interventions; identifying contacts of index patients and starting them on LTBI treatment; avoid exposing yourself to people with active TB; lead a healthy lifestyle; Get the BCG vaccination to prevent TB; begin immediate treatment for both LTBI and active TB: early screening, early treatment; ensuring proper and effective barrier nursing system by ensuring all the protective measures are adhered to; experience as a health worker, support from the government and the ministry of health, providing the LTBI drugs free of charge; patients starting on LTBI treatment.

Supplemental Figure 1. Sources of Information about TB and LTBI-Patient responses from the Post-Solutions Questionnaire

Other (Adult Contacts): Social media, community elders, outdoor poster, work.

Other (Index Patients): Community elder.
